# Integrated analysis of 14 lymphoma datasets revealed high expression of CXCL14 promotes cell migration in mantle cell lymphoma

**DOI:** 10.18632/aging.204022

**Published:** 2022-04-22

**Authors:** Dahai Liu, Fei Qi, Wei Liu, Justin Liu, Jun Wang, Dao-Qiang Lu, Yang Xun, Min-Min Chen, Xin Chen, Shu-Ting Yang, Wen-Qiao Jiao, Zong-Ye Li, Fang Liu, Hua Yang, Wen-Xing Li

**Affiliations:** 1 Foshan Stomatology Hospital and School of Medicine, Foshan University, Foshan 528000, Guangdong, China; 2 Department of Respiratory and Critical Care Medicine, Peking University Shenzhen Hospital, Futian District, Shenzhen 518036, Guangdong, China; 3 Department of Pharmacy, Anhui Medical College, Hefei 230601, Anhui, China; 4 Department of Statistics, University of California, Riverside, CA 92521, USA; 5 School of Life Science and Engineering, Foshan University, Foshan 528000, Guangdong, China; 6 Department of Biochemistry and Molecular Biology, School of Basic Medical Sciences, Southern Medical University, Guangzhou 510515, Guangdong, China; 7 Guangdong Provincial Key Laboratory of Single Cell Technology and Application, Southern Medical University, Guangzhou 510515, Guangdong, China

**Keywords:** mantle cell lymphoma, cytokines, gene expression, CXCL14, cell migration

## Abstract

Lymphoma is accompanied by the impairment of multiple immune functions. Cytokines play an important role in a variety of immune-related functions and affect the tumor microenvironment. However, the exact regulatory mechanisms between them remain unclear. This study aimed to explore the cytokines expression and function in Hodgkin's lymphoma (HL), diffuse large B-cell lymphoma (DLBCL), and mantle cell lymphoma (MCL). We performed a transcriptome integration analysis of 14 lymphoma datasets including 240 Hodgkin's lymphoma, 891 diffuse large B-cell lymphoma, 216 mantle cell lymphoma, and 64 health samples. The results showed that multiple immune functions and signal pathway damage were shared by all three types of lymphoma, and these functions were related to cytokines. Furthermore, through co-expression network and functional interaction network analysis, we identified CXCL14 as a key regulator and it affects cell chemotaxis and migration functions. The functional experiment showed that CXCL14 knockdown inhibited cell migration in MCL cell lines. This study suggested that high expression of CXCL14 may aggravate MCL via promoting cell migration. Our findings provide novel insights into the biology of this disease and would be helpful for the pathogenesis study and drug discovery of lymphomas.

## INTRODUCTION

The risk of lymphoma increases significantly with age. Aging factors such as reduced organismal functions, homeostasis imbalance, immunodeficiency, and genetic alterations can exacerbate the progression of lymphoma [1]. Hodgkin’s lymphoma (HL), a B cell-derived cancer originating in lymphocytes and involving the lymphatic system, is one of the most commonly diagnosed forms of lymphoma in the Western world. Patients are commonly diagnosed with HL in their 20 s and 30 s, and they present with supradiaphragmatic lymphadenopathy, often with systemic B symptoms [2, 3]. Hodgkin’s lymphoma also stands out as one of the most highly curable forms of cancer, with the vast majority (80% or more) of patients achieving clinical cures using modern chemo- and radiotherapy combinations [4]. Non-Hodgkin’s lymphoma (NHL) is an umbrella term referring to various closely related lymphoproliferative malignancies. According to the World Health Organization (WHO), there are more than 60 different types of cancer classified under the broader heading of NHL. Diffuse large B-cell lymphoma (DLBCL), of which more than a dozen subtypes exist, is the most common form of NHL in all countries and age groups [5, 6]. B-cell lymphomas can be classified according to their rate of growth as low grade (indolent) or high grade (aggressive). High-grade forms include DLBCL, transformed follicular lymphoma, Burkitt’s lymphoma, and lymphoblastic lymphoma. Mantle cell lymphoma (MCL) is unique in that it has features of both indolent and aggressive disease [7, 8].

Despite advances in treatments for HL and NHL, more than 20% of patients still died of disease progression. Lymphoma will cause serious damage to immune functions [9]. Immunosuppression is the most important risk factor for non-Hodgkin’s lymphoma. Various diseases and conditions can induce a state of compromised immune function which may place the patient at increased risk of NHL [8]. In addition, several immunotherapeutics have been successfully applied to HL treatment, such as Brentuximab vedotin, an antibody-drug conjugate that targets CD30 [10]. Recently published results have shown that chimeric antigen receptor (CAR) T-cell therapy is active and safe in patients with refractory large B-cell lymphoma [11]. Transcriptomic studies have also shown that impairment of immune functions can affect the prognosis of lymphoma. A previous study identified disturbed of the cell-mediated immune response, cell-to-cell signaling and interaction, and up-regulation of pathway genes involved in interleukin-12 signaling and production in macrophages and apoptosis were associated with poor prognosis of classic Hodgkin’s lymphoma (cHL) [12]. Another study showed a distinct tolerogenic host immune response between T-cell/histiocyte-rich large B-cell lymphoma (THRLBCL) and nodular lymphocyte-predominant Hodgkin’s lymphoma (NLPHL), and further identified some key regulatory genes, such as CCL8 [13]. The distinct immune status may also be the main difference between THRLBCL and NLPHL [14].

Cytokines, a broad and loose category of small proteins, play an important role in innate immune response and adaptive immune response. Cytokines can serve as metabolic hormones to provide adaptations to nutrient fluctuations, affect key metabolic processes such as glucose metabolism and lipid metabolism, eventually leading to multiple inflammations, metaflammation, and immunometabolic disorders [15]. A previous report demonstrated that Epstein-Barr virus (EBV)^+^ and EBV^-^ cHL tissues can be separated from each other by a series of gene markers, such as CXCL9, CXCL10, CCL20, and other genes that involve innate immunity and antiviral responses in EBV^+^ tumors [16]. Other studies have also reported that cytokines (including CCL3, CCL4, and CCL18) may affect the lymphoma microenvironment and patient survival both in DLBCL and MCL [17, 18]. However, there is a lack of systematic studies on these cytokines in different lymphomas. Therefore, this study aims to explore the expression and function of multiple cytokines, as well as explore key cytokines and their regulations in HL, DLBCL, and MCL through transcriptome integration analysis.

## RESULTS

### Deregulated genes overview in three types of lymphoma

The details of lymphoma datasets see our previous publication [19]. This study collected 240 HL samples, 891 DLBCL samples, 216 MCL samples, and 64 health samples. Deregulated genes in each lymphoma dataset were illustrated in Table 1. We used different fold-change (FC) thresholds to show the trend of deregulated genes in three types of lymphoma. Within the FC cutoff of 2.0 (the threshold value used in this study), we obtained 2590 up-regulated and 541 down-regulated genes, 2441 up-regulated and 903 down-regulated genes, as well as 2560 up-regulated and 515 down-regulated genes in HL, DLBCL, and MCL, respectively. Within the FC cutoff of 1.5, there were more than 4000 up-regulated genes and more than 2000 down-regulated genes in each lymphoma dataset. Within the FC cutoff of 4.0, there were also more than 600 up-regulated genes in each lymphoma dataset, however, only a handful of down-regulated genes in these lymphoma datasets. These results suggested that up-regulated genes may play a more important role in the progression of lymphoma. The top 20 differentially-expressed genes in three types of lymphoma are shown in Supplementary Table 1, all these top genes were over-expressed. Furthermore, 5 over-expressed cytokines (CCL18, CCL19, CXCL9, CXCL10, and CXCL13) were in these top 20 genes.

**Table 1:** Number of DEGs in merged datasets of the three types of lymphoma defined by different fold change cutoff values.

**Fold-change (log2)**	**Total number of DEGs (%)^1^**					
	**HL**		**DLBCL**		**MCL**	
	**Up**	**Down**	**Up**	**Down**	**Up**	**Down**
1.5 (0.58)	4583 (25.30%)	2076 (11.46%)	4199 (23.18%)	2830 (15.62%)	4566 (25.20%)	2028 (11.19%)
2.0 (1.0)	2590 (14.30%)	541 (2.97%)	2441 (13.47%)	903 (4.98%)	2560 (14.13%)	515 (2.84%)
4.0 (2.0)	668 (3.69%)	1 (0.01%)	647 (3.57%)	13 (0.07%)	653 (3.60%)	3 (0.02%)

Abbreviations: DEGs: differentially expressed genes; HL: Hodgkin's lymphoma; DLBCL: diffuse large B-cell lymphoma; MCL: mantle cell lymphoma. Up, up-regulated genes in lymphoma patients compared with controls, with log2 fold change high than the cutoffs and FDR smaller than 0.05. Down, down-regulated genes in lymphoma patients compared with controls, with log2 fold change less than the negative cutoffs and FDR smaller than 0.05. ^1^The percentage (%) = number of DEGs in the merged datasets/total number of genes in the merged datasets.

### Damaged immune functions and signaling pathways in lymphomas

**Figure 1:**
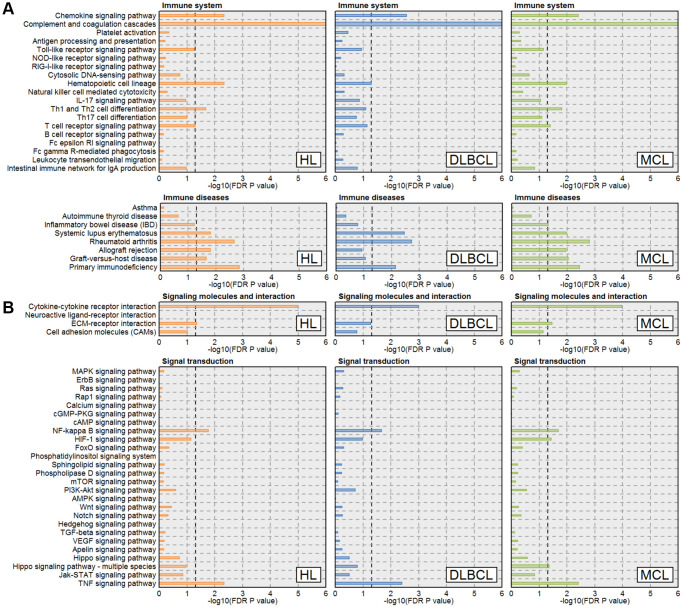
**Immune function and signaling pathway enrichment result in three types of lymphoma.** (**A**) Immune-related pathways enrichment results. (**B**) Signal transduction-related pathways enrichment results. The orange, blue and green lines represent the HL, DLBCL, and MCL, respectively. The line width indicates the enrichment percentage. The dotted line in the box indicates the significance threshold (FDR *P*-value = 0.05). Abbreviations: HL: Hodgkin's lymphoma; DLBCL: diffuse large B-cell lymphoma; MCL: mantle cell lymphoma.

KEGG pathway enrichment results of HL, DLBCL, and MCL showed in Supplementary Tables 2–4. The results showed that several immune functions and signaling pathways were significantly enriched in all three lymphomas (Figure 1). Among these immune functions, we found that chemokine signaling pathway, complement and coagulation cascades, systemic lupus erythematosus, rheumatoid arthritis, and primary immunodeficiency were enriched in all three lymphomas. Furthermore, hematopoietic cell lineage, Th1 and Th2 cell differentiation, allograft rejection, and graft-versus-host disease were enriched in HL and MCL. These results indicated that the immune functions were seriously damaged in HL, DLBCL, and MCL. Among signaling pathways, we found cytokine-cytokine receptor interaction, NF-κB signaling pathway, and TNF signaling pathway was enriched in all three lymphomas. Given the large number of cytokines involved in these pathways, it is necessary to further analyze the expression and function of cytokines in different lymphomas.

### Differentially expressed cytokines and their functions

**Figure 2:**
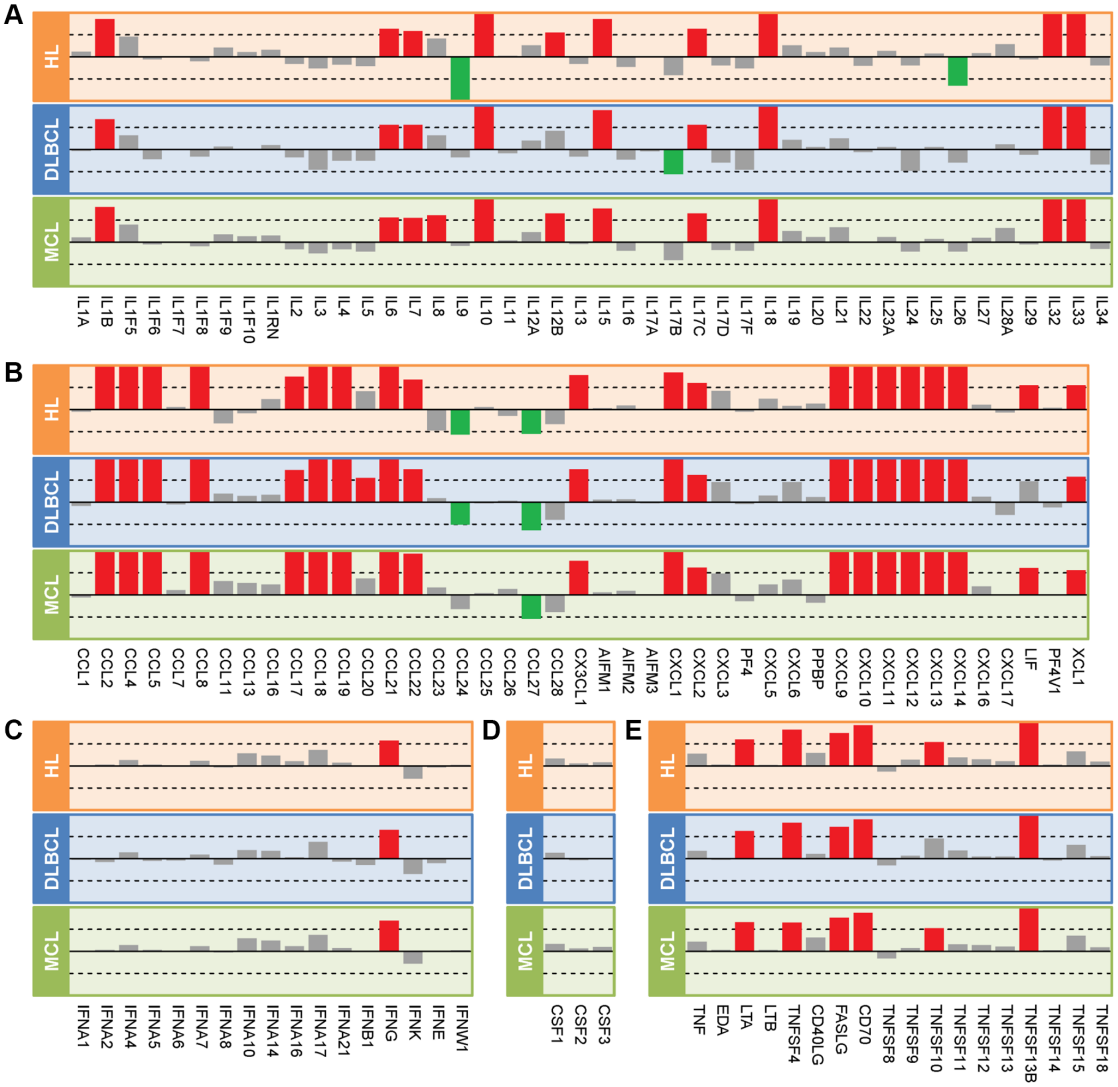
**Expression profiles of cytokines in three types of lymphoma.** (**A**) Interleukins. (**B**) Chemokines. (**C**) Interferons. (**D**) Colony stimulating factors. (**E**) Tumor necrosis factors. The length of the bar indicates the log2(fold-change) between lymphoma samples and controls. The red, green, and gray colors represent the up-regulated genes, down-regulated genes, and no change genes, respectively. The dotted line in the box indicates the log2FC = 1. Abbreviations: HL: Hodgkin's lymphoma; DLBCL: diffuse large B-cell lymphoma; MCL: mantle cell lymphoma.

**Figure 3:**
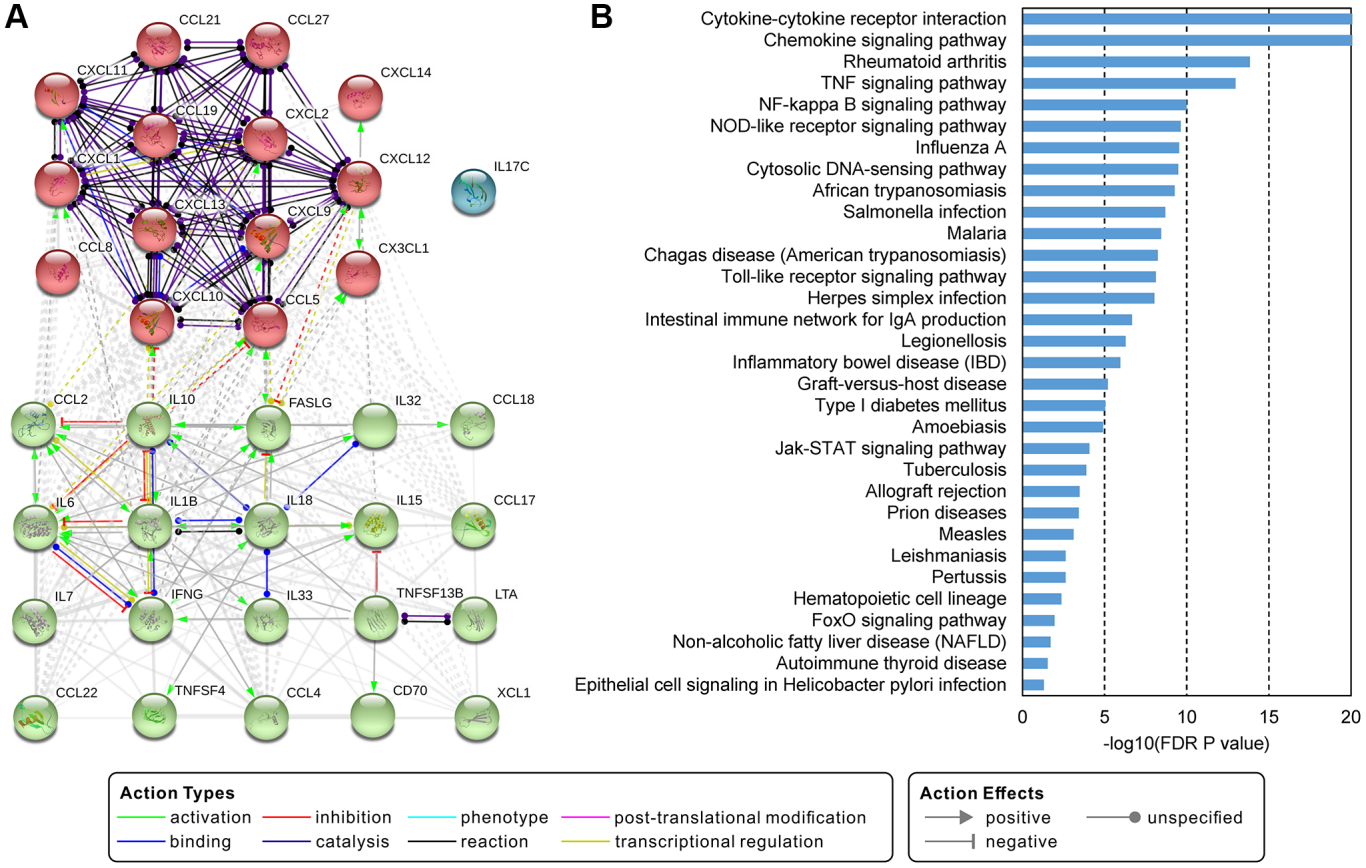
**Interactions and functions of commonly deregulated cytokines.** (**A**) Protein-protein interaction network of 35 commonly deregulated cytokines. The network uses the k-means clustering method and is clustered into 3 specified groups. (**B**) Significantly enriched KEGG pathways of genes in the network. A pathway with an FDR *P*-value less than 0.05 was considered significant.

We used the collected cytokines to match the lymphoma database and got 44 interleukins, 43 chemokines, 17 interferons, 3 colony stimulating factors, and 18 tumor necrosis factors. Expression profiles of these cytokines in HL, DLBCL, and MCL showed in Figure 2. There were 34 up-regulated cytokines in all three lymphomas. In addition, we found IL8, IL9, IL12B, IL17B, IL26, CCL20, CCL24, LIF and TNFSF10 expressed varies in three types of lymphoma. The protein-protein interaction network of the 35 commonly deregulated cytokines showed in Figure 3. By k-means clustering, we divided these cytokines into three categories. We found there were strong interactions between multiple C-C motif chemokine ligands and C-X-C motif chemokine ligands. Only IL17C has no interaction with other cytokines. There were 32 significantly enriched KEGG pathways of these commonly deregulated cytokines. Most of these pathways are associated with immune function and signaling pathways, such as cytokine-cytokine receptor interaction, chemokine signaling pathway, TNF signaling pathway, NF-κB signaling pathway, NOD-like receptor signaling pathway, and Jak-STAT signaling pathway.

### Identify key cytokines and their functions through network analysis

**Figure 4:**
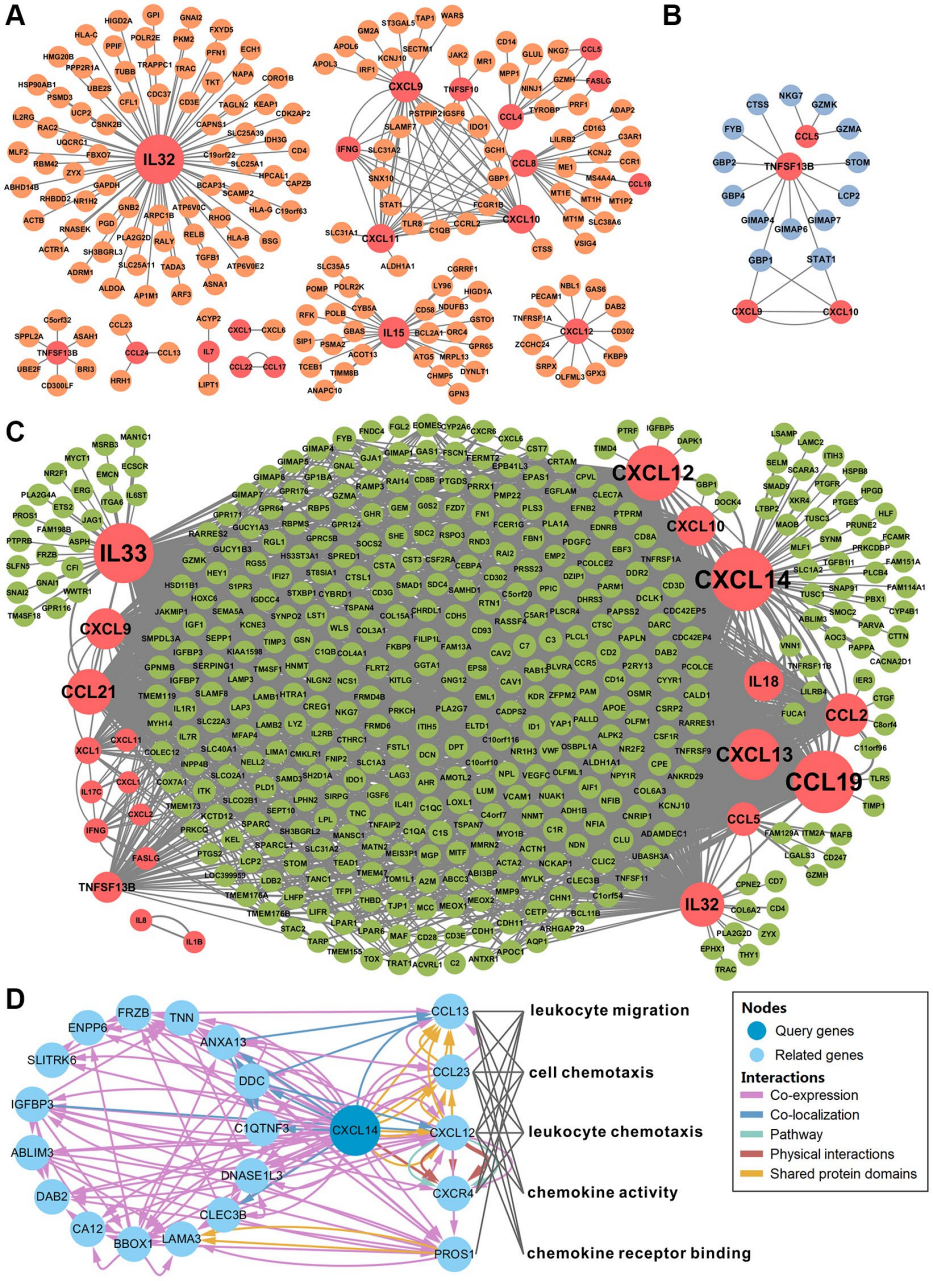
**Network analysis identified CXCL14 as a key cytokine gene.** (**A**–**C**) showed the gene co-expression networks of differentially expressed cytokines and other genes in Hodgkin's lymphoma, diffuse large B-cell lymphoma, and mantle cell lymphoma. Cytokine-gene pairs with correlation coefficients higher than 0.7 were chosen to build the network. The red circles represent the cytokines. The orange, blue and green circles represent the genes in each type of lymphoma. The size of the circle indicates the number of nodes. (**D**) Functional interaction network analysis of CXCL14. The network shows the CXCL14 related genes and their functions. Each color line represents a different interaction; the color line width indicates the weight of the interactions. There were 5 significantly enriched biological functions and the grey line indicates the gene is involved in the biological function.

Co-expression networks of deregulated cytokines and related genes in HL, DLBCL, and MCL showed in Figure 4. According to our screening criteria, there were 19 cytokines and 174 co-expressed genes in the HL network (Figure 4A), 4 cytokines and 14 co-expressed genes in the DLBCL network (Figure 4B), and 22 cytokines and 422 co-expressed genes in the MCL network (Figure 4C). The number of nodes in the DLBCL network is too small and lacks statistical power. The MCL network had the highest clustering coefficient and network centralization (Supplementary Table 5). There were no more than 100 nodes of cytokines in the HL or DLBCL network. However, there were 11 cytokines with nodes more than 100 in the MCL network (CXCL14, CCL19, IL33, CXCL12, CXCL13, CCL21, IL32, CCL2, CXCL9, CXCL10, and IL18, Supplementary Table 6). Therefore, we identified these genes as key cytokines in MCL. Among these genes, CXCL14 has the largest number of nodes and its function on lymphoma has not been reported yet. The functional interaction network showed there was physical interaction between CXCL14 and CXCR4 (Figure 4D). GO biological process enrichment analysis of these CXCL14 related genes suggested that CCL13, CCL23, CXCL12, CXCR4, and PROS1 were involved in the following 5 biological processes: leukocyte migration, cell chemotaxis, leukocyte chemotaxis, chemokine activity, and chemokine receptor binding.

### Functional experiment of CXCL14 on mantle cell lymphoma

**Figure 5:**
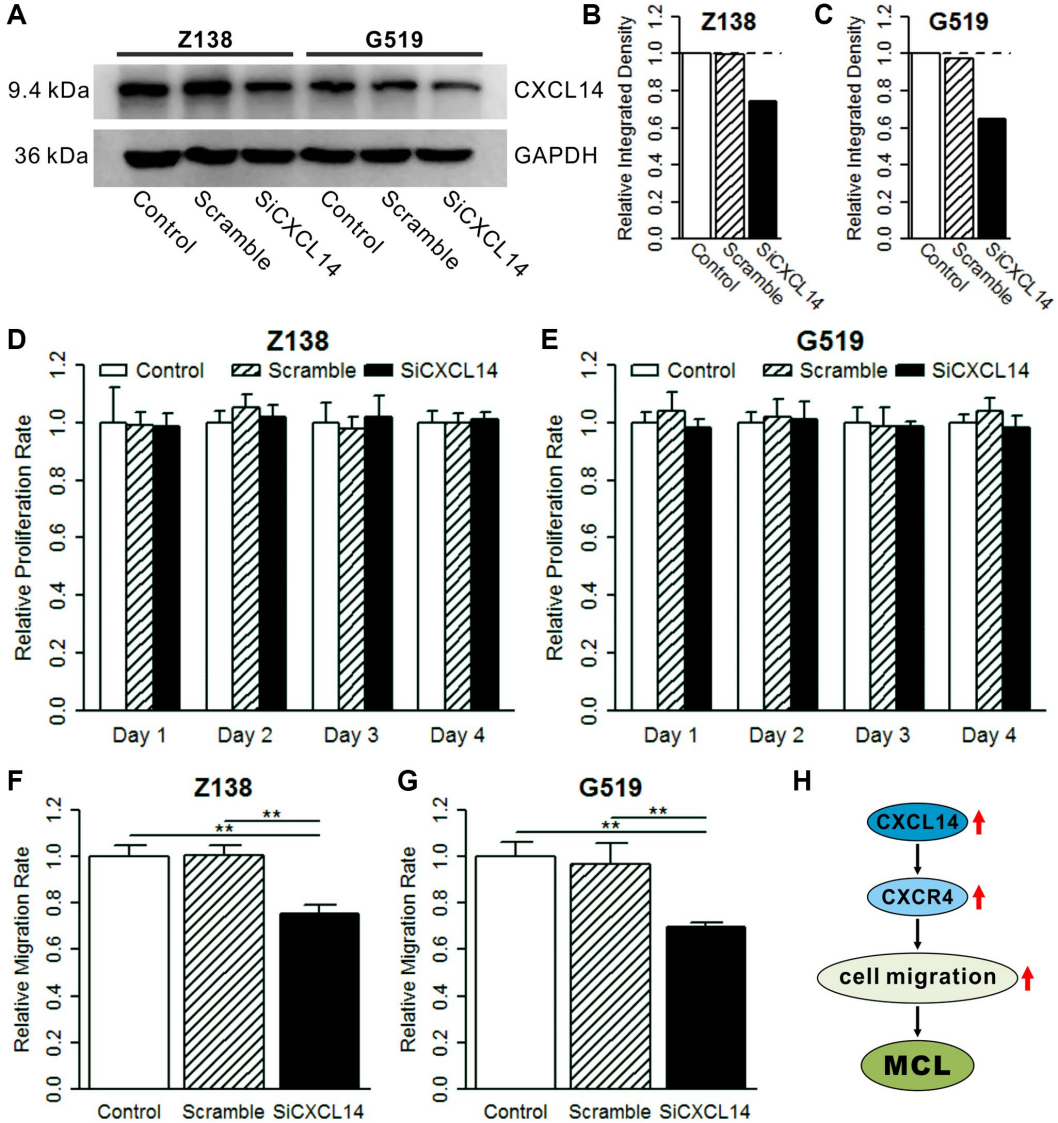
**Effect of CXCL14 on cell proliferation and migration.** (**A**) Western blot of CXCL14 in three treatment groups. Z138 and G519 cells were grown and transiently transfected with CXCL14 or negative control for 48 h and then subjected to western blotting. (**B**) Quantified band intensities in Z138 cell lines. (**C**) Quantified band intensities in G519 cell lines. The dotted line indicates the reference integrated density (control group). (**D**) Knockdown of CXCL14 on cell proliferation in Z138 cells. (**E**) Knockdown of CXCL14 on cell proliferation in G519 cells. The reference proliferation rate was defined as in the control group in each day. (**F**) Knockdown of CXCL14 on cell migration in Z138 cells. (**G**) Knockdown of CXCL14 on cell migration in G519 cells. The reference migration rate was defined as in the control group. Significance: ^***^P < 0.001, ^**^P < 0.01, ^*^P < 0.05. (**H**) High expressed CXCL14 promotes cell migration and aggravates mantle cell lymphoma.

We examined the mRNA and protein expression of CXCL14 in a normal B cell line (GM12878) and MCL cell lines (Z138 and G519). CXCL14 mRNA and protein level in Z138 were higher than in GM12878 whereas in G519 showed a lower expression (Supplementary Figure 1). To explore the function of CXCL14 on MCL, we constructed CXCL14 knockdown MCL cell lines (Z138 and G519) and performed a cell proliferation and migration assay. The results showed a lower CXCL14 protein level in the siCXCL14 group compared with control and scramble groups in Z138 and G519 cell lines (Figure 5A–5C). There was no influence of CXCL14 knockdown on cell proliferation (Figure 5D, 5E). Interestingly, the siCXCL14 group showed a significantly decreased relative migration rate compared with control and scramble groups in both two MCL cell lines (Figure 5F, 5G). The above analysis showed that high expressed CXCL14 affected cell chemotaxis and migration functions in MCL (Figure 5H). Combined with the experimental results, we speculated that high expressed CXCL14 may promote cell migration and aggravate MCL.

## DISCUSSION

Lymphomas are solid tumors of the immune system. Physiological immune checkpoint pathways are important to regulate self-tolerance, limit immune reactions, and moderate autoimmunity. Several immune checkpoint inhibitors and other cellular immunotherapies have already shown great success in HL patients [4, 20]. The most well-established risk factor for the development of NHL is immunosuppression. Patients with HIV have an increased risk of developing high-grade NHL [8]. Recently, adoptive T-cell therapy with chimeric antigen receptor T cells (CAR-Ts) has achieved clinically successful application in patients with B-cell malignancies, new data from clinical trials have demonstrated the benefits of CAR-T therapy in the NHL setting [21]. In this study, we revealed multiple damaged immune-related pathways and deregulated immune genes shared by HL, DLBCL, and MCL. Most of these pathways and genes are related to cytokine functions.

Cytokines play important roles in B-cell activation, proliferation, and apoptosis. A previous study showed dysregulated circulating cytokines (such as IL5, IL13, TNF, etc.) were correlated with B-cell non-Hodgkin lymphoma [22]. A recent study found that IL6, IL8, TNF, and other cytokines expression varies in lymphoma patients (both HL and NHL) and health populations [23]. Our results showed that 35 cytokines were consistent differentially expressed in HL, DLBCL, and MCL. Among them, over-expressed CCL18, CCL19, CXCL9, CXCL10, and CXCL13 were listed in the top 20 deregulated genes in all lymphoma datasets. CCL18 and CCL19 belong to the C-C motif chemokine ligand family. The expression of CCL18 in DLBCL was higher than the control group and patients with a higher level of CCL18 had shorter overall survival than those with lower level [24]. CCL19 and CXCL12 were previously found as chemoattractants for MCL B cells and suggested that MCL B cells were induced to migrate by CXCL12 and CCL19 [25]. CXCL9, CXCL10, and CXCL13 belong to the C-X-C motif chemokine ligand family. CXCL9 and CXCL10 are ligands for CXCR3 and attract CXCR3-expressing natural killer (NK) cells and both are up-regulated in cHL tissues. Studies showed that CXCL9 and CXCL10 may cause functional NK cell deficiencies and lead to deterioration of the tumor microenvironment of cHL [26]. In addition, high-expressed CXCL9 and CXCL10 were also found in lymphoma-associated hemophagocytic syndrome (LAHS) [27]. CXCL13 was over-production within the central nervous system (CNS) of CNS lymphoma patients, studies suggested that CXCL13 may as a potential biomarker of CNS lymphoma [28, 29].

Our data indicated that CXCL14 was critical for immune function and high expression CXCL14 may promote cell migration and aggravate MCL. CXCL14 is a non-ELR (glutamic acid-leucine-arginine) chemokine with a broad spectrum of biological activities and is expressed by a variety of immune and nonimmune cells. CXCL14 mainly contributes to the regulation of immune cell migration, also executes antimicrobial immunity [30]. The functional interaction results showed that CXCL14 interacts with CXCL12 and CXCR4. Activated CXCL12-CXCR4 axis promotes cell migration has been widely reported [31, 32]. Although CXCR4 as a receptor for CXCL14 is controversial in previous reports [33–35]. Recent evidence verified that CXCL14 binds to CXCR4 and shows a synergistic effect with CXCL12 in cancers [36, 37]. Upregulated CXCL14 expression was also found in breast implant-associated anaplastic large cell lymphoma [38], and the over-expressed CXCL14 was associated with poor survival in non-small cell lung cancer (NSCLC) patients after curative resection [39]. Recent studies indicate that CXCL14 overexpression promotes NK cell migration, cytotoxicity and infiltration [40], and is involved in thrombosis and platelet migration [41]. In addition, new evidence suggests that high expression of CXCL14 may cause endometrial cell aging [42].

In conclusion, this study identified a series of deregulated cytokines among HL, DLBCL, and MCL. These cytokines are mainly involved in immune or inflammation-related functions and signaling pathways. This study firstly reported that high expression of CXCL14 may aggravate MCL via promoting cell migration. Therefore, we suggest that CXCL14 can be used as a biomarker and a potential therapeutic target for MCL. Future studies are required to uncover its potential mechanisms.

## MATERIALS AND METHODS

### Lymphoma datasets and cytokines collection

Transcriptome datasets of HL, DLBCL, and MCL were downloaded from NCBI-GEO (https://www.ncbi.nlm.nih.gov/geo/). For detailed data inclusion and exclusion criteria, please refer to our previous work [19]. Briefly, this study collected 14 lymphoma datasets including 240 HL samples, 891 DLBCL samples, 216 MCL samples, and 64 healthy samples.

Human cytokine genes were collected from Cytokines and Cell Online Pathfinder Encyclopedia (COPE) database (http://www.cells-talk.com/index.php/page/about). According to the records of the database, cytokines are mainly divided into 5 categories: interleukins, chemokines, interferons, colony stimulating factors, and tumor necrosis factors. In total, we got 69 interleukins, 59 chemokines, 18 interferons, 3 colony stimulating factors, and 18 tumor necrosis factors from COPE (Supplementary Table 7). Then we mapped these cytokines to our datasets for subsequent analysis. The receptors of collected cytokines were obtained from previous literature [34, 35].

### Data preprocessing and differential expression analysis

R statistical software v3.4.1 (https://www.r-project.org/) was used to perform data preprocessing and differential gene expression gene analysis. The data preprocessing process has been described in detail in our previous work [19]. The empirical Bayesian algorithm in the limma package [43] was used to detect differentially expressed genes between lymphoma patients and controls. Up- and down-regulated genes were defined as a log2 transformed fold-change (logFC) ≥1 or ≤−1, respectively. A false discovery rate (FDR) corrected *P* value ≤ 0.05 was considered as significant.

### KEGG and GO enrichment analysis

Differentially expressed genes in three types of lymphoma were used to performed KEGG pathway enrichment analysis. The corresponding relationships between human genes and pathways were downloaded from the Kyoto Encyclopedia of Genes and Genomes (KEGG) database (http://www.kegg.jp/). For the formula for enrichment analysis see the previous report [44]. The enrichment percentage in each subsystem was calculated as the number of differentially expressed genes divided by the number of all genes. GO biological process enrichment analysis of filtered gene list was using BiNGO plugin in Cytoscape [45]. BiNGO is a tool to determine which Gene Ontology (GO) categories are statistically overrepresented in a set of genes or a subgraph of a biological network. We use the default parameters to perform enrichment analysis, and FDR *P*-value ≤ 0.05 was considered significantly enriched.

### Gene networks analysis

Protein-protein interaction (PPI) networks were used to explore the interactions of commonly deregulated cytokines in three types of lymphomas. We used the STRING web server (https://string-db.org/cgi/input.pl) to construct PPI networks of cytokines and other related genes. The parameter settings were: (1) the meaning of network edges was set as molecular action (line shape indicates the predicted mode of action); (2) the active interaction sources choose all types (including text-mining, experiments, databases, co-expression, neighborhood, gene fusion, and co-occurrence); (3) the minimum required interaction score was set as medium confidence of 0.4; and (4) the max number of interactors in the first shell was query proteins only, the max number of interactors in the second shell was set none or no more than 10 according to the number of query proteins. Gene co-expression networks were used to explore key cytokines. Firstly, we calculate the correlation coefficients of significantly differentially expressed cytokines and other genes in HL, DLBCL, and MCL. Then chose the cytokines-genes pairs with correlation coefficients ≥0.7 and FDR *P* values ≤ 0.05 to construct the gene co-expression networks in each lymphoma. Cytokines with the most nodes in the network were defined as key cytokines. GeneMANIA plugin in Cytoscape was used to explore the functional interaction of key cytokines and related genes [46]. Functional association networks of queried key cytokines and related genes were generated based on their relationships, such as co-expression, co-localization, pathway, physical interactions, genetic interactions, shared protein domains, and predicted. The biological functions of these genes were automatically generated and an FDR *P*-value ≤ 0.05 were considered significantly enriched.

### Cell culture

The MCL cell lines Z138 and G519 and human normal B cell line GM12878 were purchased from the Shanghai cell bank of the Chinese Academy of Sciences. The cells were maintained in RPMI 1640 medium (Gibco, Jenks, OK, USA) supplemented with 10% fetal bovine serum (Gibco, Jenks, OK, USA), 100 units/mL penicillin, and 100 μg/mL streptomycin (Gibco, Jenks, OK, USA), in a humidified atmosphere with 5% CO_2_ at 37°C.

### Transfections

The experimental group was divided into the control group (untreated Z138 and G519 cells), scramble group (mock treatment), and siCXCL14 group (CXCL14 knockdown). Z138 and G519 cells in the logarithmic growth phase were inoculated in a 6-well plate at 5 × 10^5^ cells per well. The medium was replaced with the new medium without double antibiotics. 100-pmol siRNA and 5-lL Lipofectamine 3000 were incubated at room temperature and then mixed. Consequently, the mixture was incubated for another 20 min and added to a 6-well plate. The medium was replaced after 6 h of regular cultivation. The protein expression level was detected 48 h after transfection. All siRNA duplexes targeting CXCL14 and negative control siRNAs were purchased from Ribobio Co. (Guangzhou, China), with sequences of Si CXCL14: sense, 5′-GGGUCCAAAUGCAAGUGCU-3′ and antisense, 5′-AGCACUUGCAUUUGGACCC-3′. Besides, RFect siRNA Transfection Reagent (BIO-TRAN) was used for the transfection of siRNA duplexes.

### RT-qPCR

Total RNA was extracted from individual types of Z138 and G519 cells using the RNeasy kit according to the manufacturer’s protocol (Omega Bio-Tek, Norcross, GA, USA) and reversely transcribed into cDNA using the SuperScript II reverse transcriptase (Fisher, Pittsburgh, PA, USA). The relative levels of CXCL14 mRNA transcripts in individual samples were determined using the CFX Connect (Bio-Rad, Hercules, CA, USA). The sequences of specific primers were forward 5′-GTTCTCTGAGGAACTCAAGTTTGG-3′ and reverse 5′-CTTTAAGGATCATTTGTCTCGCC-3′ for CXCL14. All samples were normalized to internal controls, and fold changes were calculated through relative quantification 2^−ΔΔCt^. The experiments were performed in triplicate.

### Western blot

Western blot was performed as previously described [47]. The primary CXCL14 antibodies is purchased from PeproTech (0.2 ug/ml, PeproTech, Inc., Cranbury, NJ, USA, Cat:500-P237). Band intensities were quantified by densitometry using ImageJ software [48].

### Cell proliferation assay

Cell proliferation assay was measured using the cell counting kit-8 (CCK8) method. 2 × 10^3^ tumor cells/well were seeded into 96-well plates and grown for 24 h, then treated as follows: control group, scramble group, and siCXCL14 group. After incubation for 24 h, 48 h, 72 h, and 96 h), 10 μl of CCK-8 solution (Dojindo Laboratories, Japan) was added to each well of the plate. The plate was incubated for an additional 4 h, and the absorbance was measured at 450 nm using a microplate reader (BioTek, Winooski, VT, USA). The percentage of cell viability (relative proliferation rate) was calculated by comparison with the control group. The experiments were performed in triplicate.

### Cell migration assay

MCL cells were treated as follows: control group, scramble group, and siCXCL14 group. After transfection of 48 h, 2 × 10^6^ cells/mL of each cell type was starved in serum-free 1640 for 12 hours at 37°C in 5% CO_2_. Migration assays were subsequently performed using Transwell chambers with 8-μm pore filters (Corning, Corning, NY, USA). Cell suspensions (2 × 10^5^ in 100 μL) were added to the upper chambers and 600 μL of medium either containing 10% FBS was added to each of the lower chambers. After transwells were incubated for 24 hours at 37°C in 5% CO_2_, the cells in each lower chamber were recovered and counted using CCK8 assay; the entire assay was repeated 3 times.

### Statistical analysis

R statistical software v3.4.1 was used for statistical analysis. The difference in cell proliferation and cell migration in the three groups were compared using the one-way analysis of variance (ANOVA). Tukey’s honestly significant difference (HSD) test was used to compared the difference between the two groups. *P* < 0.05 was considered to indicate a statistically significant difference.

## Supplementary Materials

Supplementary Figure 1

Supplementary Tables 1, 5 and 6

Supplementary Tables 2-4 and 7
