# Supplementary material for: Integrated analysis of 14 lymphoma datasets revealed high expression of CXCL14 promotes cell migration in mantle cell lymphoma
Source: Aging (Albany NY). 2022 Apr 22;14(8):3446–63. doi: 10.18632/aging.204022 (PMC9085238; doi:10.18632/aging.204022)
Supplement: Supplementary Figure 1 [file aging-14-204022-s001.pdf]

SUPPLEMENTARY FIGURE

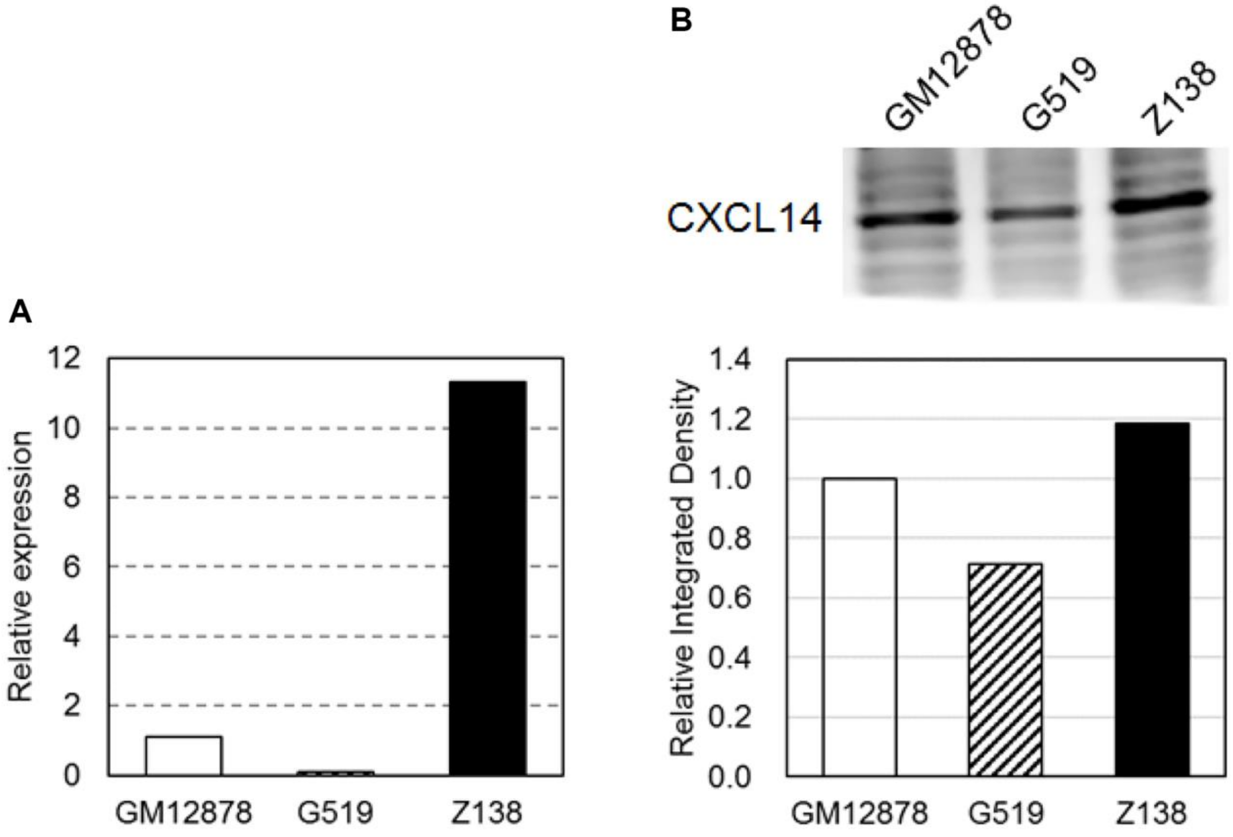

Supplementary Figure 1. CXCL14 expression in a normal B cell line (GM12878) and MCL cell lines (Z138 and G519). (**A**) RT-qPCR results of CXCL14 mRNA level in three cell lines. (**B**) Western blot results of CXCL14 mRNA level in three cell lines. The bar plot showed the quantified band intensities.
