# Supplementary material for: Integrated analysis of 14 lymphoma datasets revealed high expression of CXCL14 promotes cell migration in mantle cell lymphoma
Source: Aging (Albany NY). 2022 Apr 22;14(8):3446–63. doi: 10.18632/aging.204022 (PMC9085238; doi:10.18632/aging.204022)
Supplement: Supplementary Tables 1, 5 and 6 [file aging-14-204022-s002.pdf]

SUPPLEMENTARY TABLES

Supplementary Table 1. Top 20 differentially expressed genes in HL, DLBCL and MCL.

Hodgkin's lymphoma			Diffuse large B-cell lymphoma			Mantle cell lymphoma		
**Gene**	**logFC**	***P* value**	**Gene**	**logFC**	***P* value**	**Gene**	**logFC**	***P* value**
CXCL13	7.42	<0.001	LUM	6.98	<0.001	CXCL13	6.84	<0.001
LUM	7.33	<0.001	ADAMDEC1	6.97	<0.001	LUM	6.84	<0.001
ADAMDEC1	7.22	<0.001	LYZ	6.74	<0.001	ADAMDEC1	6.73	<0.001
LYZ	7.13	<0.001	CXCL13	6.67	<0.001	CXCL9	6.5	<0.001
COL6A3	6.63	<0.001	COL6A3	6.58	<0.001	COL6A3	6.46	<0.001
CXCL9	6.52	<0.001	CXCL9	6.47	<0.001	LYZ	6.39	<0.001
CCL19	6.44	<0.001	CCL19	6.33	<0.001	CCL18	6.15	<0.001
CCL18	6.35	<0.001	GPNMB	6.19	<0.001	CXCL10	6.08	<0.001
GPNMB	6.28	<0.001	CCL18	6.15	<0.001	CCL19	6.04	<0.001
ENPP2	6.1	<0.001	CXCL10	6.07	<0.001	COL3A1	5.98	<0.001
MMP12	6.02	<0.001	SPARCL1	6.03	<0.001	GPNMB	5.94	<0.001
CXCL10	5.99	<0.001	ENPP2	5.92	<0.001	ENPP2	5.65	<0.001
SPARCL1	5.98	<0.001	COL3A1	5.84	<0.001	MMP12	5.58	<0.001
GZMK	5.91	<0.001	VCAM1	5.66	<0.001	GJA1	5.55	<0.001
CD2	5.86	<0.001	GZMK	5.64	<0.001	MMP1	5.49	<0.001
VCAM1	5.86	<0.001	GJA1	5.56	<0.001	SPARCL1	5.49	<0.001
GJA1	5.81	<0.001	CD2	5.52	<0.001	GZMK	5.46	<0.001
COL3A1	5.8	<0.001	C1QB	5.39	<0.001	CD2	5.38	<0.001
C1QB	5.47	<0.001	COL1A2	5.38	<0.001	C1QB	5.37	<0.001
MMP9	5.41	<0.001	TIMP1	5.37	<0.001	COL1A2	5.36	<0.001

Abbreviations: HL: Hodgkin's lymphoma; DLBCL: diffuse large B-cell lymphoma; MCL: mantle cell lymphoma.

Supplementary Table 5. Co-expression network properties in three types of lymphoma.

Characteristics	HL	DLBCL	MCL
Clustering coefficient	0.072	0.111	0.642
Connected components	9	2	5
Network centralization	0.383	0.588	0.600
Network heterogeneity	2.757	1.104	3.080
Characteristic path length	2.378	1.990	2.300
Ave. number of neighbors	2.249	2.111	9.523
Number of nodes	193	18	444
Multi-edge node pairs	10	1	63

Abbreviations: HL: Hodgkin's lymphoma; DLBCL: diffuse large B-cell lymphoma; MCL: mantle cell lymphoma.

Supplementary Table 6. Node properties of cytokines in the three co-expression networks.

Lymphoma	Cytokines	Degree	Average shortest path length	Betweenness centrality	Closeness centrality	Clustering coefficient	Neighbourhood connectivity	Radiality	Topological coefficient
HL	IL32	75	1	1	1	0	1	1	0
HL	CXCL9	27	2.086	0.406	0.479	0.098	3.625	0.955	0.153
HL	IL15	27	1	1	1	0	1	1	0
HL	CXCL11	20	2.19	0.229	0.457	0.225	5.625	0.95	0.181
HL	CXCL10	20	2.379	0.111	0.42	0.242	5.625	0.943	0.179
HL	CCL8	16	2.414	0.456	0.414	0	1.375	0.941	0.075
HL	CCL4	12	2.621	0.321	0.382	0.018	1.636	0.932	0.136
HL	CXCL12	11	1	1	1	0	1	1	0
HL	IFNG	10	2.845	0.001	0.352	0.714	10.286	0.923	0.355
HL	TNFSF10	7	3.017	0.068	0.331	0.3	7.6	0.916	0.379
HL	TNFSF13B	6	1	1	1	0	1	1	0
HL	CCL24	3	1	1	1	0	1	1	0
HL	CCL5	3	3.586	0	0.279	1	6.5	0.892	0.591
HL	FASLG	2	4.517	0	0.221	0	2	0.853	1
HL	CCL22	2	1	0	1	0	1	1	0
HL	CCL17	2	1	0	1	0	1	1	0
HL	IL7	2	1	1	1	0	1	1	0
HL	CXCL1	1	1	0	1	0	1	1	0
HL	CCL18	1	4.345	0	0.23	0	2	0.861	0
DLBCL	TNFSF13B	11	1.154	0.927	0.867	0	1.364	0.986	0.182
DLBCL	CXCL10	4	2.462	0.004	0.406	0.667	3	0.867	0.75
DLBCL	CXCL9	4	2.462	0.004	0.406	0.667	3	0.867	0.75
DLBCL	CCL5	3	1	1	1	0	1	1	0

MCL	CXCL14	284	1.419	0.272	0.705	0.036	12.186	0.998	0.032
MCL	CCL19	278	1.402	0.176	0.713	0.042	13.382	0.999	0.032
MCL	IL33	261	1.465	0.208	0.683	0.041	13.195	0.998	0.034
MCL	CXCL12	228	1.567	0.11	0.638	0.046	13.723	0.998	0.035
MCL	CXCL13	200	1.605	0.057	0.623	0.068	17.005	0.998	0.041
MCL	CCL21	161	1.726	0.043	0.58	0.068	17.149	0.997	0.044
MCL	IL32	154	1.712	0.082	0.584	0.08	19.241	0.997	0.048
MCL	CCL2	147	1.76	0.042	0.568	0.079	18.633	0.997	0.047
MCL	CXCL9	130	1.747	0.048	0.573	0.125	25.06	0.997	0.06
MCL	CXCL10	124	1.763	0.05	0.567	0.115	22.92	0.997	0.055
MCL	IL18	121	1.814	0.054	0.551	0.094	20.478	0.997	0.052
MCL	CCL5	80	2.081	0.05	0.48	0.097	17.5	0.996	0.055
MCL	TNFSF13B	61	2.456	0.034	0.407	0.066	12.776	0.995	0.075
MCL	XCL1	32	2.584	0.007	0.387	0.198	18.321	0.994	0.121
MCL	IFNG	16	2.998	0.009	0.334	0.132	7.357	0.993	0.187
MCL	FASLG	7	3.126	0.001	0.32	0.267	7	0.992	0.311
MCL	CXCL11	7	2.688	0.005	0.372	0.3	48.8	0.994	0.375
MCL	IL17C	6	1	1	1	0	1	1	0
MCL	IL8	2	1	0	1	0	1	1	0
MCL	IL1B	2	1	0	1	0	1	1	0
MCL	CXCL2	1	1	0	1	0	1	1	0
MCL	CXCL1	1	1	0	1	0	1	1	0

Abbreviations: HL: Hodgkin's lymphoma; DLBCL: diffuse large B-cell lymphoma; MCL: mantle cell lymphoma.
